# A c.726C>G (p.Tyr242Ter) nonsense mutation-associated with splicing alteration (NASA) of *WDR45* gene underlies β-propeller protein-associated neurodegeneration (BPAN)

**DOI:** 10.1016/j.heliyon.2024.e30438

**Published:** 2024-05-04

**Authors:** Qiongling Peng, Ying Cui, Jin Wu, Lianying Wu, Jiajia Liu, Yangyun Han, Guanting Lu

**Affiliations:** a Department of Child Healthcare, Shenzhen Bao'an Women's and Children's Hospital, 56 Yulyu Road, Bao'an District, Shenzhen, 518000, China; b Department of Blood Transfusion, The First Affiliated Hospital of Xi'an Jiaotong University, No. 277 West Yanta Road, Xi'an, 710061, China; c Laboratory of Translational Medicine Research, Department of Pathology, Affiliated Deyang People's Hospital of Sichuan Traditional Medical University, No. 103 First Section of Taishanbei Road, Jingyang District, Deyang, 618000, China; d Deyang Key Laboratory of Tumor Molecular Research, No. 103 First Section of Taishanbei Road, Jingyang District, Deyang, 618000, China; e Sichuan Clinical Medical Research Center for Neurological Diseases, No. 103 First Section of Taishanbei Road, Jingyang District, Deyang, 618000, China

**Keywords:** BPAN, WDR45, Nonsense mutation, Pre-mRNA splicing, Nonsense-mediated mRNA decay, Nonsense-associated splicing alteration

## Abstract

Neurodegeneration with brain iron accumulation (NBIA) is a clinically and genetically heterogeneous disease characterized by increased iron deposition in the basal ganglia and progressive degeneration of the nervous system in adulthood. However, in early childhood, there were no characteristic features to perform early diagnosis. In our study, a female child exhibited global developmental delay, intellectual disability, and febrile seizure without other distinct clinical phenotypes. Through whole exome sequencing (WES), a *de novo* nonsense mutation (c.726C > G, p. Tyr242Ter) of WDR45 gene was identified in this child. She was finally diagnosed as β‐propeller protein-associated neurodegeneration (BPAN), one of the recently identified subtypes of NBIA. This mutation could act as a premature stop codon (PSC) which rendered the mutated transcripts to be degraded by nonsense-mediated mRNA decay (NMD), leading to decreased levels of PSC-containing mRNAs. Additionally, through mini-gene splicing assays, this mutation could result in an unprecedented novel transcript with the exon 9 of *WDR45* excluded by nonsense-associated splicing alteration (NASA). Transcriptome sequencing (RNA-seq) on total RNAs from PBMCs of the trio revealed three types of alternative splicing events in the patient. Further research implied that downregulation of iron transport genes (*TFRC*, *TFR2*, *SCARA5*) might be the underlying mechanism for the iron accumulation in patients with deficient WDR45. This is the first report about NASA happening in *WDR45*. It implies that nonsense mutations approximal to splicing sites could affect the disease pathogenesis through more than one molecular mechanism and should be taken into consideration when conducting genetic counseling.

## Highlights

• A nonsense mutation c.726C > G (p.Tyr242Ter) was identified in WDR45.
• The mutation generated a novel transcript excluding exon 9 through NASA.
• Iron metabolism-related genes were abnormally regulated by deficient WDR45.

## 1 Introduction

Neurodegeneration with brain iron accumulation (NBIA) is a group of neurodevelopmental disorders characterized by progressive brain iron deposition, which predominantly occurs in the basal ganglia [1]. It has been reported that the pathogenic process of NBIA is progressive, comprising a broad phenotypic spectrum with a distinct two-phase clinical phenotype: childhood-onset developmental delay, intellectual disability, and epilepsy; followed by adulthood-onset dystonia, parkinsonism, dementia, and especially iron accumulation in the basal ganglia [[2], [3], [4], [5]]. To date, eight genes have been identified to be responsible for a spectrum of NBIAs: PANK2 for NBIA1 [6], *PLA2G6* for NBIA2 [7,8], *FTL* for NBIA3 [9,10], *C19orf12* for NBIA4 [11], *WDR45* for NBIA5 [3,12], *COASY* for NBIA6 [13,14], *REPS1* for NBIA7 [15] and *CRAT* for NBIA8 [15]. The abnormal brain iron accumulation in the basal ganglia was also observed in other disorders, such as Kufor-Rakeb syndrome (KRS), Aceruloplasminemia (ACEP), Spastic paraplegia 35 (SPG35), and Jaberi-Elahi syndrome (JABELS), which were caused by mutated *ATP13A2* [16], *CP* [17], *FA2H* [18], *GTPBP2* [19], respectively. It seems that NBIA is varied clinically and genetically, which presents big challenges to achieve a diagnosis solely through clinical phenotypes [20]. With the increasing application of high-throughput sequencing techniques such as whole exome sequencing (WES), whole genome sequencing (WGS), and transcriptome sequencing (RNA-seq), it's very helpful for the early diagnosis of patients without specific phenotypes.

In our current project, a female child presented with global developmental delay, intellectual disability, and febrile seizures at 1 year and 2 months old. Through array-CGH and WES, a *de novo* nonsense mutation (c.726C > G, p. Tyr242Ter) was identified in the coding sequence (CDS) of the *WDR45* gene. Combined with the molecular analysis and clinical phenotypes, the patient was finally diagnosed as BPAN. Notably, during the very early stages of childhood with BPAN, it's difficult to distinguish from other neurodevelopmental disorders, because brain iron deposition was undetectable through MRI till progressing into adulthood. Therefore, molecular testing should be an indispensable auxiliary diagnosis for BPAN.

The human *WDR45* gene is located on the X-chromosome, encoding a protein of 360 amino acids, with the seven *N*-terminal WD-like repeats formed a β-propeller-like shape [21]. WDR45 is expressed ubiquitously, with a relative abundancy in the hippocampus [22]. Mutations in WDR45 were first reported to be responsible for one type of NBIA in 2012 [2]. Till now, about 150 WDR45 variants have been identified [23]. The nonsense mutation (c.726C > G, p. Tyr242Ter) identified in our patient is located in the sixth WD-like repeat of WDR45. The mutation could function as a premature stop codon (PSC) to render the mutated transcripts be degraded by nonsense-mediated mRNA decay (NMD).

In order to analysis if this PSC could affect the function of WDR45 in other known way, bioinformatic and experimental analysis were performed. The mutation was three bases proximal to the canonical splicing donor site of intron 9. This proximity might influence the canonical pre-mRNA splicing of *WDR45* according to our bioinformatic analysis. Through minigene splicing assay, a novel type of transcript skipping the exon 9 was generated by c.726C > G nonsense-associated splicing alteration (NASA) to eliminate the PSC-harboring transcripts. Transcriptome sequencing (RNA-seq) was conducted to verify the existence of this splicing event. Bioinformatic analysis identified some abnormally-expressed iron metabolism-related genes which might be affected by WDR45 deficiency.

### 1.1 This report represents a significant contribution to the understanding of WDR45 mutations, as it is the first documented case about a nonsense mutation influencing the pre-mRNA splicing of WDR45, in addition to its conventional role as a premature stop codon. Results

#### 1.1.1 Patient description

The proband, a three-year-old girl, was born naturally to a non-consanguineous couple. She has a one-year-old unaffected brother. The gestational period was 39 weeks, and her birth weight was 3.00 kg. Regular prenatal examinations during pregnancy showed no identifiable abnormalities. Slight neonatal jaundice was appeared but did not require medical intervention. Concerns arose when the parents noticed gross motor delay at 6 months and lack of steady head control. The diagnosis of global developmental delay was made at 1 year and 3 months old. Rehabilitation started at 1 year and 8 months old, but yielded limited progress.

At the latest follow-up at 3 years old, her height and weight were within the average range for her age (96 cm and 16 kg). Neurological examination revealed no dystonia or nystagmus. She could walk independently but struggled with running, and independent spoon-feeding. She had limited understanding and impaired speech abilities. Neuropsychological assessments using CNBS-R2016 and ABAS-II at 3 years old indicated a mild to moderate global developmental delay. CNBS-R2016 full-scale developmental quotient (DQ) was 53, with subscales (gross motor, fine motor, adaptive behavior, language, and personal-social) ranging from 47 to 56. ABAS-II overall adaptive function score was 63, with composite scores in social, conceptual, and practical skills. Blood biochemical analysis and metabolic disease screening at 2 years old showed no abnormalities. MRI scans at 20 and 36 months old revealed no abnormal signals, except for bilateral small arachnoid cysts. A febrile seizure occurred at 1 year and 2 months old, but no convulsive seizures happened afterward. Electroencephalogram (EEG) showed no abnormal discharge signals. No abnormalities were revealed by neural electrophysiological examinations at 20 months old (fVEP and ABR).

In order to give a quick diagnosis of the disease, a Phenotype Profile Search was carried out at Human Phenotype Ontology (HPO) [24] using the 10 clinical phenotypes of the patient, such as intellectual disability, global developmental delay, language impairment, poor speech, hypotonia, difficulty running, abnormal social understanding, febrile seizure, feeding difficulties and arachnoid cyst. Ten types of inheritable diseases were obtained, with autosomal recessive intellectual developmental disorder-64 (MRT64) and autosomal dominant intellectual developmental disorder-46 (MRD46) covered all of the inputting phenotypes, which was caused by mutations in *LINGO1* and *KCNQ5* genes, respectively (Fig. 1). The patient was still not exactly diagnosed based on the clinical phenotypes.

**Fig. 1:**
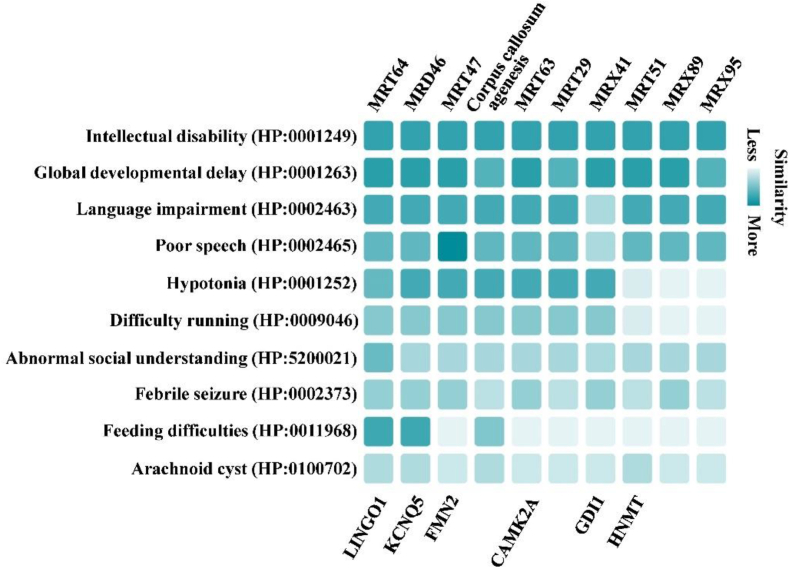
Phenotype profile searching.

#### 1.1.2 A *de novo* nonsense mutation of *WDR45* identified by Trio-WES

**Fig. 2:**
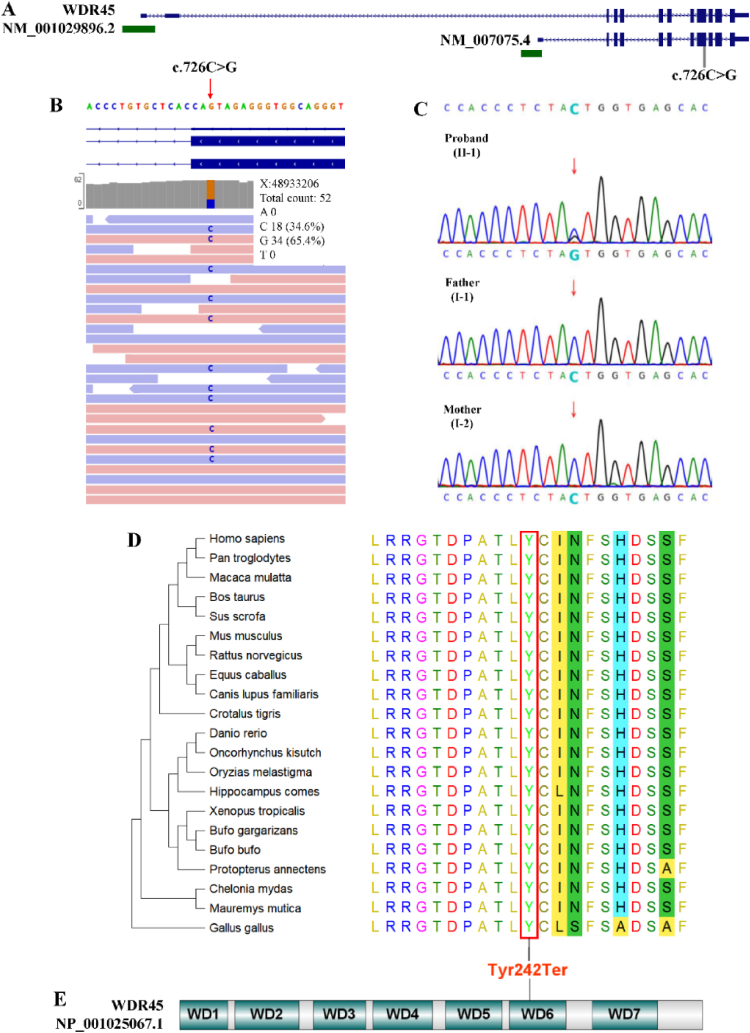
**A nonsense mutation identified in the patient**. A) Genomic structure of *WDR45* gene; B) c.726C > G identified by WES; C) Sanger sequencing; D) Evolutionary conservation analysis; E) Protein structure of WDR45.

In order to have an exact diagnosis of the patient and find out the molecular factors of the disease, G-banding chromosome karyotype analysis of PBMCs were performed and no evident chromosomal abnormalities revealed. Array-based Comparative Genomic Hybridization analysis (Array-CGH) did not detect any disease-associated microduplications or microdeletions. Trio-WES identified a nonsense mutation (c.726C > G, chrX:48, 933, 206, hg19) in exon 9 of the *WDR45* gene (NM_007075.3) exclusively in the proband (Fig. 2A and B). Sanger sequencing confirmed this mutation, with mutant reads accounting for 65.4 % of the totally covered sequences (Fig. 2C). The mutation introduced a premature stop codon (PSC) at position 242, leading to the replacement of an evolutionarily conserved Tyrosine (p.Tyr242Ter) (Fig. 2D) in the sixth WD-repeat domain (blade) of the WDR45 protein (Fig. 2E). The mutation has not been annotated in the NCBI dbSNP database and was absent in large-cohort databases, including 1000Genome (n = 2504), ExAC (n = 60,706), NHLBI GO-ESP (n = 6503), gnomAD (n = 125,748), and TOPMED (n = 206,000). Following the ACMG guidelines, this mutation was classified as “Pathogenic' (PVS1+PS2+PM2). It has been reported that premature stop codon could render the mutated transcripts to be degraded by nonsense-mediated mRNA decay (NMD) to decrease the levels of PSC-containing mRNAs [25].

#### 1.1.3 Novel potential splicing sites generated by c.726C > G

**Fig. 3:**
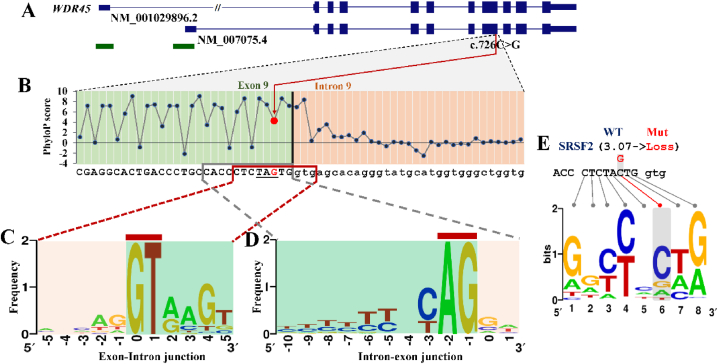
**Genomic features around the mutation.** A) Genomic structure of *WDR45* gene; B) Conservation analysis; C) Consensus motifs for the Exon-intron junction; D) Consensus motifs for the Intron-exon junction; E) Motif of Exonic splicing enhancer analysis for SRSF2.

Despite as a nonsense mutation, c.726C > G was found to be only three nucleotides away from the canonical splicing donor site (c.728 + 1, chrX:48, 933, 203) within intron 9 (Fig. 3A and B). Notably, the exon-intron junction demonstrated high conservation, as indicated by PhyloP base-wise conservation scores (Fig. 3B). Analysis suggested that the mutant 726G allele could potentially create a novel exon-intron junction with GT as the boundary (Fig. 3C) and a novel intron-exon junction with AG as the boundary (Fig. 3D). This implies that c.726C > G might be potentially detrimental to the consensus splicing regulatory motifs around the ninth exon-intron junction.

As per the analysis conducted by ESEFinder (version 3.0), the c.726C > G transversion was predicted to lead to the loss of an exonic splicing enhancer (ESE) motif, which was likely bound by the serine/arginine-rich (SR) splicing factor 2 (SRSF2) (Fig. 3E–Table 1). SRSF2 is one of several ESE-binding factors responsible for recognizing exonic sequences during pre-mRNA splicing. Additionally, this transversion might impact the binding affinities of SRSF5 for two putative ESE motifs. If any of these potential SR protein binding motifs proves to be functional, the mutation could be detrimental to the canonical pre-mRNA splicing of *WDR45*. Therefore, the c.726C > G alteration not only has the potential to lead to the degradation of the mutant mRNAs through NMD, but might also disrupt the canonical splice site, resulting in the generation of aberrantly spliced isoforms.

**Table 1:** Effects of 726C > G on ESE motifs.

SR proteins	Motif threshold	WT (score)	Mutant (score)
SRSF2	2.38	CTCTA**C**TG (3.07)	Loss
SRSF5	2.67	CCTCTA**C** (3.26)	CCTCTA**G** (4.11)
SRSF5	2.67	CTA**C**TGg (5.41)	CTA**G**TGg (3.69)

#### 1.1.4 Nonsense-associated pre-mRNA splicing of *WDR45* revealed by mini-gene splicing assay

**Fig. 4:**
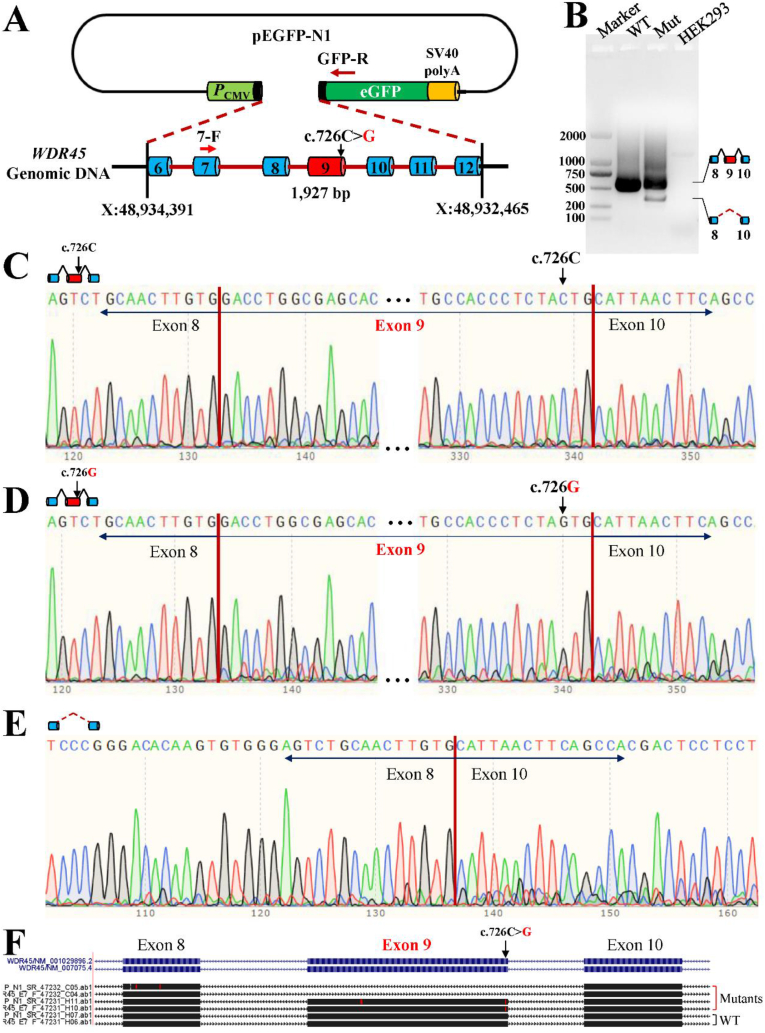
**726C** > **G (Tyr242Ter) disrupted pre-mRNA splicing of *WDR45***. A) Diagram of mini-gene assay constructs for *WDR45* gene; B) Agarose electrophoresis; C) Sanger sequencing for the wild-type transcripts; D) Sanger sequencing for the long Mut transcripts; E) Sanger sequencing for the short Mut transcripts; F) Alignment for sequenced PCR products. WT, Wild type; Mut, Mutant. 7-F, forward primer in exon 7, GFP-R, reverse primer in GFP.

In order to experimentally test the possibility of abnormal splicing caused by the nonsense mutation (c.726C > G), a 1927 bp genomic region containing exon 6 to 9 plus introns of *WDR45* was cloned into the multiple cloning site (MCS) of pEGFP-N1 vector (Fig. 4A). The mutation (Mut, 726G) was introduced by site-directed mutagenesis. This construct was then transfected into HEK293 cells for a mini-gene splicing assay. Agarose electrophoresis of the PCR products revealed a single band of 600 bp in the wild-type sample and two distinct bands in the Mut sample. Specifically, the Mut sample displayed a 600 bp band, nearly identical to the wild-type transcript except for the mutated nucleotide c.726G, and a shorter 400 bp band, indicating intragenic splicing between exon 8 and 10, resulting in the exclusion of exon 9 (Fig. 4B, C, D, E). An unclear smear above the 600 bp band in Mut samples was attributed to unspecific amplification. Upon aligning all Sanger-sequenced bands against the WDR45 reference, three types of isoforms were identified (Fig. 4F). It clearly revealed that a novel splicing event with the exclusion of exon 9 of *WDR45* was produced by the identified c.726C > G nonsense-associated pre-mRNA splicing alteration (NASA) to eliminate PSC-containing transcripts.

#### 1.1.5 Splicing patters of *WDR45* revealed by transcriptome sequencing

**Fig. 5:**
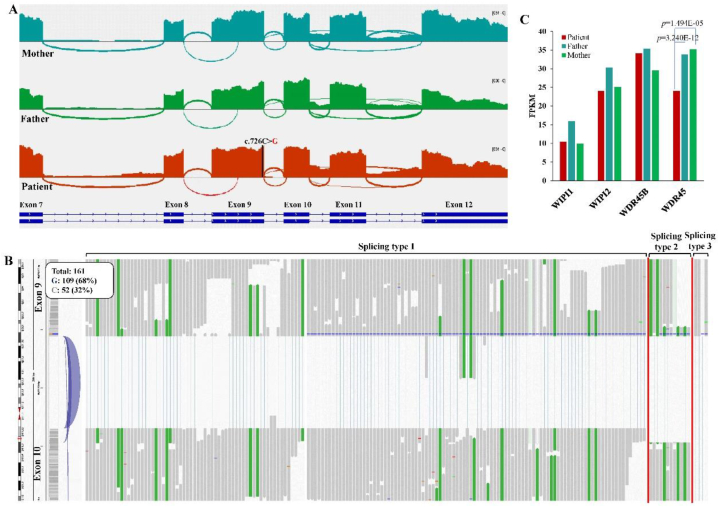
Splicing patterns of WDR45 identified by RNA-seq. A) Splicing around exon 9; B) Three types of splicing events between exon 9 and exon 10; C) Relative expression of four WIPI genes.

Although abnormal splicing was identified by mini-gene splicing assay, the splicing patters of WDR45 in the patient was unknown. Transcriptome sequencing (RNA-seq) was conducted on total RNAs from PBMCs of the trio, revealing three types of alternative splicing events involving the c.726C > G mutation (Fig. 5A and B). The first type of splicing (90.48 %, 152 reads) occurred between the canonical splicing sites of intron 9, with 64.47 % (98/152) containing the mutation (c.726G) and the remaining 35.53 % (54/152) capable of producing functional wild-type WDR45 protein. The second type (7.14 %, 12 reads) involved a cryptic splicing acceptor (chrX:48, 933, 113), 12bp downstream of the canonical splicing acceptor site, resulting in a putative protein lacking 4 amino acids. The third type (2.38 %, 4 reads) resulted from splicing between exon 9 and exon 12, removing exon 10 and exon 11. Unexpectedly, these splicing events were also observed in the father and mother with a similar expression level (Fig. 5A). Notably, the novel splicing with the exclusion of exon 9 was not identified in the patient, which was inconsistent with the results obtained by the mini-gene splicing assay.

Intriguingly, a missense mutation (c.281 A > G, p. Lys94Arg) was identified in the father, present in 14.93 % (reads ratio = 30/201) of the sequenced reads covering the region. This mutation is recorded in dbSNP (rs2065040047) and annotated as “Likely Benign' (PM2+BP1+BP4) according to the ACMG guidelines. Unexpectedly, two sequenced reads (0.78 %, reads ratio = 2/255) containing the nonsense mutation (c.726C > G) were also identified in the father. It's implied that there might be an unknown molecular mechanism in the patient for the propensity to induce the appearance of the nonsense mutation (c.726C > G).

#### 1.1.6 Bioinformatic analysis of the differentially expressed genes

It has been reported that there are four members of the WIPI subfamily (WIPI1, WIPI2, WDR45 and WDR45B) with similar functions in the process of autophagy. The expression levels of these four genes were examined, revealing a significantly lower level of WDR45 in the patient compared to both the father (*p* = 3.24E-12) and mother (*p* = 1.49E-05) (Fig. 5C). This might be attributed to the rapid mRNA degradation of mutated WDR45 by NMD.

**Fig. 6:**
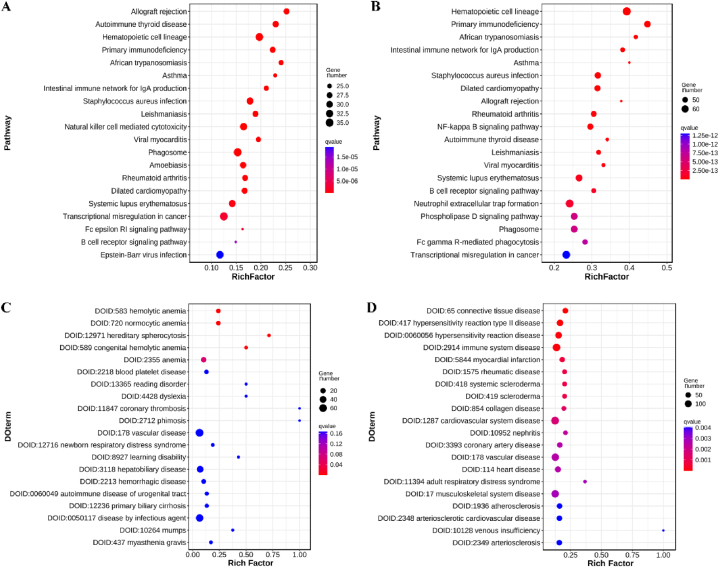
Bioinformatic analysis of the differentially expressed genes. A) pathway analysis in P/M; B) pathway analysis in P/F; C) DO analysis in P/M; D) DO analysis in P/F.

Genes often interact to carry out their functions, and pathway-based analysis provides a more comprehensive understanding of their biological roles. The Kyoto Encyclopedia of Genes and Genomes (KEGG) is a major public pathway-related database [26]. Pathway enrichment analysis was performed to identify significantly enriched metabolic pathways or signal transduction pathways in Differentially Expressed Genes (DEGs). The top 20 pathways with the lowest Q value were selected for two groups: patient vs. mother (P/M, Fig. 6A, Table S1) and patient vs. father (P/F, Fig. 6B–Table S2).

In both groups, most of the DEGs were enriched in pathways related to the immune system, such as allograft rejection, autoimmune thyroid disease, hematopoietic cell lineage, primary immunodeficiency, asthma, and others. Disease Ontology (DO) enrichment analysis was conducted to identify significantly enriched human disease DO terms in DEGs. In the P/M group, only four DO terms were significantly enriched, including hemolytic anemia (q = 0.000713), normocytic anemia (q = 0.000713), hereditary spherocytosis (q = 0.001079), and congenital hemolytic anemia (q = 0.001363) (Fig. 6C–Table S3). In the P/F group, 62 significantly enriched DO terms were identified, with connective tissue disease (q = 0.000006), hypersensitivity reaction type II disease (q = 0.000021), hypersensitivity reaction disease (q = 0.000065), immune system disease (q = 0.000065), and myocardial infarction (q = 0.000663) ranking among the top five (Fig. 6D–Table S4).

#### 1.1.7 Iron metabolism-related genes were abnormally regulated

**Fig. 7:**
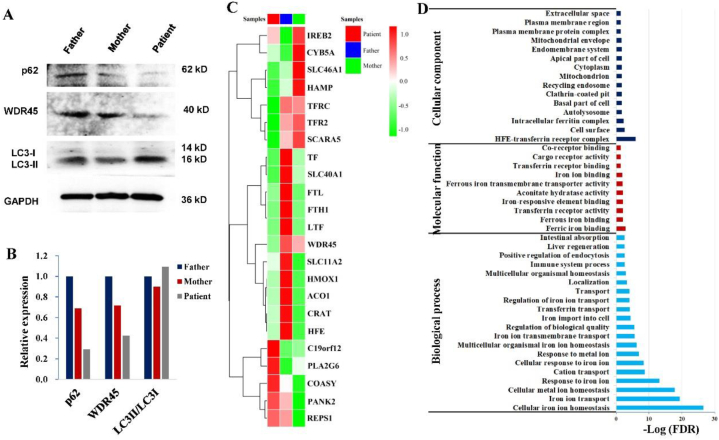
Genes involving iron metabolism. A) Western blot of three proteins for autophagy; B) Quantification of the tree proteins for autophagy; C) Expression of genes involving iron metabolism; D) Gene ontology analysis of the selected proteins involving iron metabolism.

WDR45 was proved to be critical for the formation of the autophagosome [27,28] and mutations in this gene resulted in the impairment of autophagy process [29]. Two crucial autophagy markers, p62 and LC3, along with WDR45, were investigated by Western blot in PBMCs of the trio (Fig. 7A). Although the expression of LC3 remained almost the same in the three samples, the levels of p62 and WDR45 were significantly decreased in the patient (Fig. 7B). It's implied that the autophagy might be impaired in the patient.

Considering the involvement of WDR45 in autophagy progression and its association with the pathogenesis of neurodegeneration with excessive brain iron accumulation, the expression of genes related to iron metabolism was compared between the patient and her parents (Fig. 7C). In addition to *WDR45*, three other genes responsible for iron transmembrane transportation (*TFRC*, *TFR2* and *SCARA5*) were also significantly downregulated in the patients. Among the reported genes for NBIAs (Table S5), *C19orf12*, *PLA2G6*, *COASY*, *PANK2* and *REPS1* were significantly overexpressed in the patient (Fig. 7C). Gene Ontology (GO) analysis was performed for the selected iron metabolism genes and found that four genes (*HFE*, *TF*, *TFRC*, and *TFR2*) were significantly enriched in the “HFE-transferrin receptor complex' (FDR = 1.21E-06). In “molecular function,' the genes clustered in “ferric iron binding,' “ferrous iron binding,' “transferrin receptor activity,' and other related functions. For “biological process,' the top five GO terms included “Cellular iron ion homeostasis' (FDR = 2.74E-27), “Iron ion transport' (FDR = 4.88E-20), “Cellular metal ion homeostasis' (FDR = 1.60E-18), “Response to iron ion' (FDR = 6.45E-14), and “Cation transport' (FDR = 1.73E-09) (Fig. 7D). It seemed that iron metabolism-related genes could be abnormally regulated by mutated WDR45.

## 2 Discussion

It has been reported that the clinical manifestations of NBIA could be typically divided into two phases. The initial phase is characterized by global developmental delay, intellectual disability, and/or epilepsy during childhood. In the second phase, individuals may experience progressive dystonia, parkinsonism, and dementia in adulthood [2,4,20,30]. MRI findings in early childhood are commonly reported as normal, till in adulthood, hyperintense signals in T1 and low signals in T2 could be observed in the basal ganglia, indicating abnormal iron accumulation [4,30]. Since the clinical and genetic heterogeneities of NBIA vary greatly, early diagnosis is a huge challenge for clinicians and genetic consultants just based on clinical phenotypes. With the rapid advancement of high-throughput sequencing techniques such as WES, WGS and RNA-seq, their application for detecting molecular factors proves to be valuable for the early clinical diagnosis of NBIA during childhood. This enables timely intervention and appropriate treatment before the condition further deteriorates.

In this study, a 3-year-old girl presented with developmental delay, intellectual disability, and experienced a febrile seizure at 1 year and 2 months old. There were no typical features that could distinguish NBIA from other neurodevelopmental disorders. MRI only revealed a cystoid shadow in the occipital cisterna magna and the anterior edge of the left temporal pole, along with slightly dilated bilateral ventricles. The phenotype profile searching at Human Phenotype Ontology (HPO) using the ten observable clinical phenotypes obtained no specific abnormalities. It's difficult to carry out an etiological diagnosis of the patient based on the current clinical phenotypes. Through trio-WES, a *de novo* nonsense mutation (c.726C > G, p. Tyr242Ter) was identified in the WDR45 gene. Pathogenic mutations of WDR45 have been attributed to the pathogenesis of brain iron accumulation-5 (NBIA5), which was also called as BPAN [2,30]. This mutation has been previously reported in two female children, one diagnosed with BPAN [31] and the other with severe infantile-onset developmental and epileptic encephalopathy [32]. Therefore, the molecular analysis played an important role in confirming the diagnosis of BPAN in the early phase for this patient.

The nonsense mutation (c.726C > G), introducing a premature stop codon (PSC), could potentially lead to the degradation of mutant transcripts by NMD [33,34]. This mutation located only three nucleotides away from the canonical splicing donor site (c.728 + 1) of intron 9 and might disrupt an exonic-splicing-enhancer (ESE) motif bound by SRSF2, thereby affecting the pre-mRNA splicing of WDR45. Building on our previous findings that variants proximal to intron/exon boundaries could result in complex aberrant splicing [35], this nonsense mutation (c.726C > G) near splicing site could potentially disrupt putative ESE motifs and cause aberrant pre-mRNA splicing. Mini-gene splicing assays confirmed the generation of a novel splicing variant excluding exon 9 caused by the nonsense mutation (c.726C > G). If translatable, it could produce a truncated protein with 173 amino acids (p.Val172fs × 1). This phenomenon was referred to as nonsense-associated splicing alteration (NASA) [36,37]. NASA could increase the existence of alternatively-spliced transcripts that skipped PSC-containing exons. Transcripts containing premature stop codons (PSCs) might be poisonous as they could encode potentially deleterious shortened proteins. To avoid the generation of truncated proteins, NMD was activated to decrease the level of PSC-containing transcripts. It seemed that cells might evolve positive and negative quality-control mechanisms to limit the presence of PSC-containing mRNAs.

Apart from nonsense mutations, other single nucleotide polymorphisms (SNPs) or rare neutral variants within exonic splicing enhancers or silencers could also influence the patterns or efficiency of pre-mRNA splicing. This variability in splicing might contribute to the diverse clinical phenotypes and mutation penetrance observed in diseases caused by different mutations in the same gene [35]. This phenomenon raised a big challenge to assess the penetrance or contribution of a given mutation to the pathogenesis of inheritable diseases.

According to the WES data, the c.726C > G mutation was detected in 34.60 % of the sequenced reads, a finding corroborated by Sanger sequencing. This observation suggested the existence of the mutation in a portion, not all of the PBMCs of the female patient, indicating a “mosaic' pattern. Subsequent RNA-seq analysis revealed the mutation in 38.69 % (65/168) of the sequenced reads. In females, who possess two copies of X chromosomes, one copy undergoes random inactivation to maintain balanced expression of X-linked proteins comparable to males. Consequently, the X chromosome containing the mutant allele could be active in some cells, producing non-functional proteins, while it might be inactive in others, where functional proteins were produced by the wild-type X chromosome. Since the types and proportions of X-inactivated cells in various systems can vary significantly, the severity of clinical symptoms might differ markedly. Inferred from RNA-seq, it is suggested that the c.726C > G mutated X chromosome was active in the PBMCs of this patient. Surprisingly, two sequenced reads with the same mutation (c.726C > G) were identified in the father's PBMCs, which might be attributed to sequencing errors. Alternatively, there might be an unknown mechanism in the patient that led to a higher prevalence of cells carrying the mutation compared to the father.

It has been reported that WDR45 played a crucial role in various biological pathways, including autophagy, iron storage, ferritin metabolism and endoplasmic reticulum (ER) homeostasis [22,29,[38], [39], [40]]. Genes related to iron metabolism and NBIA were analyzed extensively. Except for *WDR45*, the patient exhibited decreased expressions of three other genes crucial for iron transmembrane transport (*TFRC*/*TFR1*, *TFR2*, and *SCARA5*). TFRC/TFR1 is the receptor for iron-bound Transferrin at the surface of plasma membrane responsible for transportation of iron. It has been reported that TFRC knockout mice exhibited impaired development of erythrocytes and nervous system which resulted in early embryonic lethality [41]. Mutated TFRC in human could cause systemic immunodeficiency [42]. TFR2 is a homolog to TFRC. Mutations in the TFR2 gene could induce increased intestinal iron absorption by increased iron uptake leading to tissue iron overload [43,44]. SCARA5 could bound serum ferritin and then stimulated its endocytosis from the cell surface with consequent iron delivery [45]. The abnormal intracellular iron transport might worsen the severity caused by mutated WDR45. Five genes associated with NBIA (*C19orf12, PLA2G6, COASY, PANK2*, and *REPS1*) were increased in the patient, but these genes were unrelated to iron metabolism. Four of them (*C19orf12*, *PLA2G6*, *COASY*, and *PANK2*) are located in mitochondria, with *PANK2* and *COASY* coding for enzymatic proteins essential for coenzyme A (CoA) production. C19orf12 and PLA2G6 are involved in lipid metabolism, membrane integrity, and mitochondrial function [46]. REPS1, involved in endocytosis and vesicle transport [15], was also upregulated. Notably, these five genes were associated with the mitochondrion, suggesting a pivotal role for mitochondria in NBIA pathogenesis. However, the link between upregulated NBIA-associated genes and abnormal brain iron metabolism remains unclear. Some NBIA genes, including those involved in lipid metabolism (C19orf12, PLA2G6, and COASY) and autophagy (WDR45), have been shown to play essential roles in cellular iron homeostasis. Additionally, genes responsible for lipid metabolism are crucial for ferroptosis [[47], [48], [49]]. These findings suggested a potential relationship between autophagy, lipid metabolism, and cellular iron metabolism in NBIA.

Certainly, functional determination of detected variants in disease-related genes is crucial to classify them as pathogenic, low-penetrance, or neutral variants. This approach is essential for improving diagnostic accuracy, estimating disease progression, and facilitating timely symptomatic treatment. Accurate variant classification enhances our understanding of the molecular mechanisms in underlying diseases, allowing for more effective and personalized approaches to treat patients.

## 3 Conclusion

In summary, due to the highly variable two-phase phenotypes of NBIA, genetic testing is extremely important for an early diagnosis of diseases which were very helpful for timely treatments. In addition to function as premature stop codons (PSCs), some nonsense mutations near splicing sites could disrupt putative regulatory exonic splicing motifs to affect pre-mRNA splicing. This might be a putative correction mechanism to generate alternatively-spliced novel transcripts to skip PSCs. This study represents the first report of nonsense-associated splicing alteration (NASA) in WDR45. This phenomenon might be an underlying mechanism for the low penetrance of some mutations and the variable phenotypes observed in certain diseases.

## 4 Materials and methods

### 4.1 Sample collection

The study was conducted in strict adherence to the ethical guidelines outlined in the Code of Ethics of the World Medical Association, specifically the Declaration of Helsinki, governing experiments involving humans. Approval for the study was granted by the Ethical Committees of Shenzhen Bao'an Women's and Children's Hospital. Additionally, written informed consents were obtained from the parent of the patient, ensuring compliance with ethical standards.

Peripheral venous blood samples were collected from both the patient and her parent for the purpose of the study. Genomic DNAs were subsequently extracted using the TIANamp Blood DNA Kit (DP348, Tiangen Biotech, Beijing, China), following the manufacturer's instructions.

### 4.2 Molecular analysis of the mutation

Chromosome karyotype analysis was conducted on PBMCs to identify any potential chromosomal abnormalities. Subsequently, oligonucleotide array-CGH was executed using the Fetal DNA Chip (Version 1.2), following established procedures as outlined in our previous reports [50,51]. WES was conducted for the trio, consisting of the proband and her parent, at MyGenostics. Each sample achieved a sequencing depth of approximately 100*x*. Reads obtained from the sequencing were mapped to the human reference genome (UCSC hg19). The analysis of the WES data followed methodologies outlined in our previous reports, ensuring consistency and comparability with established protocols (Supplementary figure) [50,51]. In brief, the preprocessing of data involved obtaining clean reads through the removal of adaptors and low-quality reads from the raw data in fastq format, utilizing Btrim [52]. Subsequently, variant calling on the trimmed and cleaned WES data was conducted using GATK (Genome Analysis Toolkit) (https://gatk.broadinstitute.org/hc/en-us). Functional annotation for the GATK-called variants was performed by the software ANNOVAR [53]. Variants with a Minor Allele Frequency (MAF) exceeding 0.1 % or those identified as synonymous single nucleotide variants (SNVs) were excluded from further consideration. SNVs leading to splicing alterations, frameshifts, stop gain, or stop loss were retained for subsequent analysis. Information regarding the location, type, and conservation of the identified mutations was retrieved from multiple public databases, including the UCSC Genome Browser, dbSNP, ClinVar, 1000Genome, ExAC, TOPMED, and gnomAD. Additionally, nonsynonymous single nucleotide variants (SNVs) underwent further analysis by being submitted to PolyPhen-2 (Polymorphism Phenotyping v2) [54] and PROVEAN (Protein Variation Effect Analyzer) [55] for functional prediction. The clinical significance of the identified variants was interpreted following the guidelines provided by the American College of Medical Genetics and Genomics (ACMG) classification [56]. The authenticity of the identified candidate variants was verified through Sanger sequencing, a reliable method for confirming genetic variations. Additionally, to gain insights into the evolutionary conservation of the WDR45 protein, protein sequences from WDR45 in 21 different animals were obtained from NCBI GenBank. The alignment of these protein sequences was performed using the integrated ClustalW alignment algorithms within MEGA 11. Specific parameters for the alignment process, including gap opening penalty, gap extension penalty for pairwise alignment, and multiple alignment, were set at 10.00, 0.10, and 10.00, 0.20, respectively. The Delay divergent cutoff was established at 30 % [57]. To assess the evolutionary conservation of the sequences around the ninth exon-intron junction of WDR45, PhyloP base-wise conservation scores were obtained from the “Conservation' track in the UCSC Genome Browser. Furthermore, ESE Finder was employed to analyze the potential disruption of exonic splicing enhancer motifs around the identified nonsense mutation in the WDR45 gene. This analysis helps understand the impact of the mutation on splicing regulatory elements, providing insights into potential alterations in mRNA processing [58].

### 4.3 Mini-gene splicing assays

A 1,927bp genomic region (chrX:48, 932, 465–48,934,391, hg19), encompassing eight exons (from exon 6 to 12, plus introns) of the WDR45 gene, was synthesized and cloned into the multiple cloning site (MCS) of the pEGFP-N1 plasmid for the mini-gene splicing assay. Introducing the nonsense mutation (c.726C > G) was achieved through site-directed mutagenesis. Human embryonic kidney 293 (HEK293) cells were cultured in high-glucose Dulbecco's Modified Eagle Medium (DMEM) medium (FI101-01, TransGen, Beijing, China) supplemented with 5 % fetal bovine serum (FBS) in 5 % CO_2_. The wild-type (Wt) and mutated (Mut) constructs were transfected into HEK293 cells using TransIntro EL/PL Transfection Reagent (FT231-02, TransGen), respectively. After 24 h of transfection, cells were harvested, and lysed using 5 mL TransZol (ET101-01, TransGen). Total RNAs were extracted and reverse transcribed into complementary DNAs (cDNAs) using TransScript Reverse Transcriptase (AT101-02, TransGen). The cDNAs were amplified by polymerase chain reaction (PCR) with paired primers, subjected to electrophoresis on a 1.5 % agarose gel (120 V for 25 min), and visualized using the ChemiDoc XRS + Gel Imaging System (Bio-Rad, Hercules, California, USA).

### 4.4 Western blot analysis

Proteins were extracted from PBMCs using RIPA lysis buffer (50 mM Tris pH 7.4, 150 mM NaCl, 1 % NP 40, 1 % sodium deoxycholate, 1 mM PMSF, and a protease inhibitor cocktail). Subsequently, the proteins underwent electrophoresis by SDS-PAGE and were transferred onto PVDF membranes. For western blotting, primary antibodies included *anti*-GAPDH (1:5000, 60,004-4-lg, Proteintech), *anti*-LC3 (1:1000; 3868 S, CST), *anti*-p62 (1:10000, 66184-1-Ig, Proteintech), and *anti*-WDR45 (1:1000, PA5-121,118, Thermofisher). Secondary antibodies employed for western blotting were anti-rabbit IgG, horseradish peroxidase (HRP)-linked (1:10,000; Proteintech), or anti-mouse IgG, HRP-linked (1:10,000; Proteintech).

### 4.5 Transcriptome sequencing (RNA-seqs) for the trio

Total RNA was isolated using the Trizol reagent kit following the manufacturer's protocol. The quality of RNA was evaluated using an Agilent 2100 Bioanalyzer (Agilent Technologies, Palo Alto, CA, USA) and confirmed through RNase-free agarose gel electrophoresis. mRNA enrichment was performed using Oligo (dT) beads from total RNA. Subsequently, the enriched mRNA underwent fragmentation using a fragmentation buffer and was reverse-transcribed into cDNA with random primers. Second-strand cDNA synthesis was carried out using DNA polymerase I, RNase H, and dNTP. The resulting cDNA fragments were purified using the QiaQuick PCR extraction kit (Qiagen, Venlo, The Netherlands), subjected to end repair, poly(A) addition, and ligation to Illumina sequencing adapters. Ligation products were size-selected via agarose gel electrophoresis, PCR amplified, and sequenced using the Illumina HiSeq 2500 platform at Gene Denovo Co. (Guangzhou, China).

### 4.6 Bioinformatics analysis

Clean reads were obtained following filtration by fastq [59] to remove adapters, reads with more than 10 % of unknown nucleotides (N), and reads containing over 50 % of low-quality bases (Q-value≤20). Ribosomal RNAs (rRNAs) were eliminated through alignment to the ribosomal RNA database [60] using Bowtie 2 (version 2.2.8) [61]. The resultant clean reads were mapped to the reference genome using HISAT2. 2.4 [62] with default parameters and the option "-rna-strandness RF."

Mapped reads from each sample were assembled using StringTie v1.3.1 [63] in a reference-based approach. For each transcription region, the expression abundance and variations were quantified using the Fragments Per Kilobase of transcript per Million mapped reads (FPKM) value. Differential expression analysis was conducted using DESeq2 software [64] between two distinct groups and edgeR [65] between two samples. Genes with a false discovery rate (FDR) parameter below 0.05 were considered differentially expressed.

## Fundings

This work was supported by fundings from the Natural ScienceFoundation of Sichuan (2023NSFSC0601), Key 10.13039/100006190Research and Development Project of Deyang science and Technology Bureau (2021SZ003, 2020SZZ085), Special Fund for Incubation Projects of Deyang People's Hospital (FHG202004) and Hospital Special Project for “Xinglin Scholar' of 10.13039/501100008402Chengdu University of Traditional Chinese Medicine (YYZX202258).

## Data availability

Transcriptome sequencing data will be made available at National Genomics Data Center (NGDC) with an accession: PRJCA025575.

## CRediT authorship contribution statement

**Qiongling Peng:** Resources, Funding acquisition. **Ying Cui:** Formal analysis, Data curation. **Jin Wu:** Methodology. **Lianying Wu:** Data curation. **Jiajia Liu:** Data curation. **Yangyun Han:** Writing – review & editing, Supervision. **Guanting Lu:** Writing – review & editing, Writing – original draft, Visualization, Formal analysis, Conceptualization.

## Declaration of competing interest

The authors declare that they have no known competing financial interests or personal relationships that could have appeared to influence the work reported in this paper.

## Figures and Tables

**figs1:**
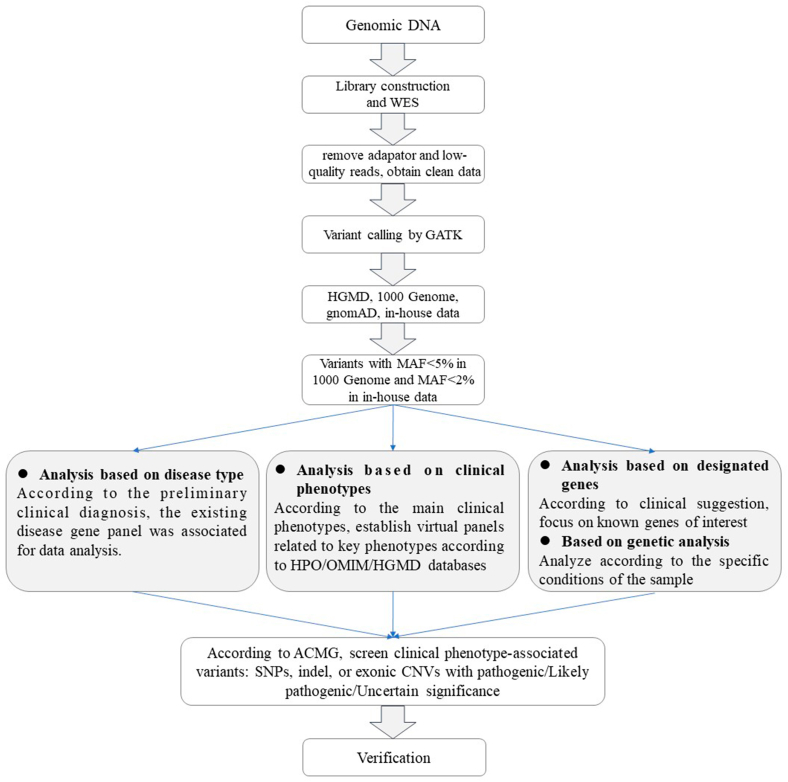


## Abbreviations

BPAN: beta-propeller protein-associated neurodegeneration
NBIA: neurodegeneration with brain iron accumulation
HSD: Hallervorden-Spatz disease
KRS: Kufor-Rakeb syndrome
ACEP: Aceruloplasminemia
SPG35: Spastic paraplegia 35
XLD: X-linked dominant inheritance
WES: whole exome sequencing
WGS: whole genome sequencing
RNA-seq: transcriptome sequencing
CDS: coding sequence
Array-CGH: array-based comparative genomic hybridization
NASA: nonsense-associated splicing
NMD: nonsense-mediated mRNA decay
PSC: premature stop codon
MRI: magnetic resonance imaging
EEG: Electroencephalogram
HPO: Human Phenotype Ontology
ESE: exonic splicing enhancer
KEGG: Kyoto Encyclopedia of Genes and Genomes
DEG: differentially expressed gene
PBMC: peripheral blood mononuclear cell
GATK: Genome Analysis Toolkit
ER: endoplasmic reticulum
MAF: minor allele frequency
SNV: single nucleotide variant
ACMG: American College of Medical Genetics and Genomics
DQ: developmental quotient
MRT: intellectual developmental disorder
SRSF2: serine/arginine-rich (SR) splicing factor 2
ESE: exonic splicing enhancer
WT: wild-type
MCS: multiple cloning site
GO: Gene Ontology
SNPs: single nucleotide polymorphisms
ER: endoplasmic reticulum
CoA: coenzyme A
SNVs: single nucleotide variants
FBS: fetal bovine serum
DMEM: Dulbecco's Modified Eagle Medium
HEK293: human embryonic kidney 293
cDNAs: complementary DNAs
PCR: polymerase chain reaction
rRNAs: Ribosomal RNAs
FPKM: Fragments Per Kilobase of transcript per Million mapped reads
FDR: false discovery rate
